# Supplementary material for: An Evaluation of a Virtual Food Safety Program for Low-Income Families: Applying the Theory of Planned Behavior
Source: Foods. 2022 Jan 26;11(3):355. doi: 10.3390/foods11030355 (PMC8834591; doi:10.3390/foods11030355)
Supplement: Supplementary file 1 [file foods-11-00355-s001.zip › foods-1535953-supplementary.pdf]

Supplemental Material: Additional Results Tables

Table S1. Barriers addressed throughout the program using directly and indirectly interventions

Barrier	Program activity	**How was it address?** (directly or indirectly)[a]
**Knowledge**		
Low-income individuals lack knowledge about some food safety practices.	• Information on the core four food safety practices was provided.<br>• Topics were discussed using "Questions and Answers" format.	Directly
**Geographical factors**		
Low-income individuals have to travel farther to the nearest supermarket.	• Discussions about the importance of temperature and time were held.<br>• Discussions about the temperature and time perishable food could be left at room temperature were held.	Indirectly
Supermarkets, grocery stores, convenience stores in low-income neighborhoods might have higher microbial loads in their produce and/or surfaces.	• Information on the core four food safety practices was provided.	Indirectly
**Lack of food safety tools**		
The low-income individuals may lack of a cooking and/or refrigerator thermometer and might not have multiple cutting boards at home.	• A cooking thermometer was provided (ThermoWorks 600D Super-Fast Waterproof Pocket Thermometer).<br>• A refrigerator/freezer thermometer was provided (ThermoWorks RT615 Digital Fridge/Freezer Thermometer).<br>• Information about the safe cooking temperature of food and cross-contamination was provided.	Directly and indirectly
**Cultural factors**		

There is a lack of food safety education specific to ethnic dishes.	• One activity was developed based on the participants’ selection of a dish, including (1) tres leches, (2) brownies, (3) meatloaf, or (4) Tacos.	Directly and indirectly

[a]In this educational program, the barriers of low-income home food handlers were addressed directly and indirectly. Directly refers that the barriers were addressed straightforwardly by activities inside the program. Indirectly refers that the barriers were not addressed straightforwardly, but the program's content helped to think of ways to address those barriers.

Table S2. Frequencies and percentages of correct answers for knowledge questions before and after the intervention

Questions	**English Program** (n = 30)		**Spanish Program** (n = 30)		**Total** (n = 60)	
	**Pre-survey** n (%)	**Post-survey** n (%)	**Pre-survey** n (%)	**Post-survey** n (%)	**Pre-survey** n (%)	**Post-survey** n (%)
What are the recommended hand washing steps?	28 (93)	30 (100)	23 (77)	29 (97)[a]	51 (85)	59 (98)[a]
How can bacteria spread while preparing foods?	30 (100)	30 (100)	29 (97)	30 (100)	59 (98)	60 (100)
What is the recommended way to store a large pot of soup?	16 (54)	30 (100)[a]	19 (63)	30 (100)[a]	35 (58)	60 (100)[a]
Where should raw meat and poultry be stored in the refrigerator?	27 (90)	29 (97)	24 (80)	29 (97)[a]	51 (85)	58 (97)[a]
What is the maximum time food (meat, poultry, cut fruit) should be left at room temperature?	22 (73)	29 (97)[a]	20 (67)	28 (93)[a]	42 (70)	57 (95)[a]
What is the recommended refrigerator temperature?	17 (57)	30 (100)[a]	14 (47)	26 (87)[a]	31 (52)	56 (93)[a]
What is the recommended internal temperature of thoroughly cooked chicken?	20 (67)	28 (93)[a]	10 (34)	23 (77)[a]	30 (50)	51 (85)[a]
What is the recommended internal temperature of thoroughly	14 (47)	25 (83)[a]	6 (20)	20 (67)[a]	20 (34)	45 (75)[a]

cooked ground beef?

[a]The frequency of correct answers between the pre- and post- survey of the knowledge question was significantly different, $P \leq 0.05$.

Table S3. Participants' mean scores of statements based on the Theory of Planned Behavior constructs, behavior change intention, and self-reported safe food practice behavior, before and after the intervention

**Statements**	**English Program** (n = 30)		**Spanish Program** (n = 30)		**Total** (n = 60)	
	**Pre-Survey (**Mean ± SD)	**Post-Survey** (Mean ± SD)	**Pre-Survey (**Mean ± SD)	**Post-Survey** (Mean ± SD)	**Pre-Survey (**Mean ± SD)	**Post-Survey** (Mean ± SD)
Attitude towards the behavior[a]						
It is important to keep my cooking areas clean.	5.00 ± 0.00	5.00 ± 0.00	5.00 ± 0.00	4.90 ± 0.40	5.00 ± 0.00	4.95 ± 0.29
It is important to use separate cutting boards to prevent cross-contamination.	4.60 ± 0.72	4.93 ± 0.25[d]	4.73 ± 0.52	4.97 ± 0.18[d]	4.67 ± 0.63	4.95 ± 0.22[d]
It is important to use a cooking thermometer to measure the temperature of ground beef.	3.97 ± 0.81	4.93 ± 0.25[d]	3.87 ± 1.14	4.80 ± 0.41[d]	3.92 ± 0.98	4.87 ± 0.34[d]
It is important to check refrigerator and freezer temperatures to keep perishable foods safe.	4.60 ± 0.67	5.00 ± 0.00[d]	3.87 ± 1.41	4.87 ± 0.43[d]	4.23 ± 1.16	4.93 ± 0.31[d]
It is important to divide a large pot of soup into shallow containers for storage.	4.03 ± 0.85	4.93 ± 0.25[d]	4.30 ± 0.95	4.87 ± 0.43[d]	4.17 ± 0.91	4.90 ± 0.35[d]
It is important to ensure my children wash their hands before meals.	4.93 ± 0.25	4.97 ± 0.18	4.07 ± 1.36	5.00 ± 0.00[d]	4.50 ± 1.07	4.98 ± 0.13[d]
I believe my children are NOT at risk for developing a	3.37 ± 1.54	3.27 ± 1.80	3.90 ± 1.32	3.60 ± 1.63	3.63 ± 1.45	3.43 ± 1.71

foodborne illness.						
PBC[a]						
I am confident in my ability to keep my cooking areas clean.	4.47 ± 0.73	4.97 ± 0.18[d]	4.73 ± 0.45	4.97 ± 0.18[d]	4.60 ± 0.62	4.97 ± 0.18[d]
I am confident in my ability to use separate cutting boards to prevent cross-contamination.	4.40 ± 0.89	4.80 ± 0.48	4.63 ± 0.89	4.83 ± 0.38	4.52 ± 0.89	4.82 ± 0.43[d]
I am confident in my ability to use a cooking thermometer to measure the temperature of ground beef.	4.30 ± 0.84	4.97 ± 0.18[d]	3.97 ± 1.13	4.80 ± 0.48[d]	4.13 ± 1.00	4.88 ± 0.37[d]
I am confident in my ability to check refrigerator and freezer temperatures to keep perishable food safe.	4.43 ± 0.77	4.90 ± 0.31[d]	4.30 ± 0.84	4.93 ± 0.25[d]	4.37 ± 0.80	4.92 ± 0.28[d]
I am confident in my ability to divide a large pot of soup into shallow containers.	4.27 ± 0.78	4.90 ± 0.31[d]	4.50 ± 0.73	4.87 ± 0.43[d]	4.38 ± 0.76	4.88 ± 0.37[d]
I am confident in my ability to ensure my children wash their hands before meals.	4.93 ± 0.25	4.97 ± 0.18	4.80 ± 0.76	4.97 ± 0.18	4.87 ± 0.57	4.97 ± 0.18
I am confident in my ability to prepare foods that do not make my children sick.	4.87 ± 0.35	4.97 ± 0.18	4.63 ± 0.61	5.00 ± 0.00[d]	4.75 ± 0.51	4.98 ± 0.13[d]

Subjective norm[a]						
My family/friends believe it is important to keep cooking areas clean.	4.53 ± 0.68	4.73 ± 0.52	4.77 ± 0.43	4.87 ± 0.43	4.65 ± 0.57	4.80 ± 0.48
My family/friends believe it is important to use separate cutting boards to prevent cross-contamination.	3.87 ± 1.22	4.33 ± 0.76	4.37 ± 0.89	4.47 ± 0.86	4.12 ± 1.09	4.40 ± 0.81
My family/friends believe it is important to take the temperature of ground beef when cooking.	3.33 ± 1.15	4.37 ± 0.81[d]	3.43 ± 1.55	4.27 ± 0.87[d]	3.38 ± 1.35	4.32 ± 0.83[d]
My family/friends believe it is important to check refrigerator and freezer temperatures to keep perishable foods safe.	4.07 ± 1.08	4.53 ± 0.63[d]	4.17 ± 0.91	4.57 ± 0.77[d]	4.12 ± 0.99	4.55 ± 0.70[d]
My family/friends believe it is important to divide a large pot of soup into shallow containers for storage.	3.53 ± 1.17	4.37 ± 0.85[d]	4.43 ± 0.82	4.60 ± 0.67	3.98 ± 1.10	4.48 ± 0.77[d]
My family/friends believe(s) it is important to ensure my	4.47 ± 0.97	4.77 ± 0.50	4.90 ± 0.31	4.86 ± 0.43	4.68 ± 0.75	4.82 ± 0.47

children wash their hands before meals.						
My family/ friends believe my children are NOT at risk for developing a foodborne illness.	3.33 ± 1.47	3.47 ± 1.70	3.83 ± 1.34	3.60 ± 1.65	3.58 ± 1.42	3.53 ± 1.67
My family/ friends believe the way I prepare foods will not make my children sick.	4.77 ± 0.77	4.53 ± 0.94	4.63 ± 0.67	4.77 ± 0.77	4.70 ± 0.72	4.65 ± 0.86
Behavior change intention[a]						
I will keep my cooking areas clean.	4.87 ± 0.35	4.97 ± 0.18	4.90 ± 0.31	4.93 ± 0.25	4.88 ± 0.32	4.95 ± 0.22
I will use separate cutting boards to prevent cross-contamination.	4.40 ± 0.67	4.80 ± 0.48[d]	4.80 ± 0.55	4.97 ± 0.18	4.60 ± 0.64	4.88 ± 0.37[d]
I will use a cooking thermometer to measure the temperature of ground beef.	4.07 ± 0.83	5.00 ± 0.00[d]	4.07 ± 1.11	4.83 ± 0.38[d]	4.07 ± 0.97	4.92 ± 0.28[d]
I will check the refrigerator and freezer temperatures to keep perishable foods safe.	4.50 ± 0.57	4.97 ± 0.18[d]	4.57 ± 0.63	4.97 ± 0.18[d]	4.53 ± 0.60	4.97 ± 0.18[d]
I will ensure my children wash their hands before meals.	4.93 ± 0.25	4.93 ± 0.25	4.90 ± 0.31	5.00 ± 0.00	4.92 ± 0.28	4.97 ± 0.18
I will follow recommended food practices to the reduce the risk of my	4.97 ± 0.18	5.00 ± 0.00	4.90 ± 0.40	5.00 ± 0.00	4.93 ± 0.31	5.00 ± 0.00

children getting sick.						
Self-reported safe food practice behavior[b]						
I keep my cooking areas clean.	4.57 ± 0.50	4.83 ± 0.38[d]	4.63 ± 0.49	4.83 ± 0.38[d]	4.60 ± 0.49	4.83 ± 0.38[d]
I wash my hands after handling raw meat, poultry, or seafood.	4.93 ± 0.25	4.97 ± 0.18	4.93 ± 0.25	4.87 ± 0.43	4.93 ± 0.25	4.92 ± 0.33
I cut meat and fruit on the same cutting board after rinsing with water[c]	4.03 ± 1.45	4.50 ± 1.17[d]	3.17 ± 1.67	3.40 ± 1.87	3.60 ± 1.61	3.95 ± 1.64
I use a cooking thermometer to determine the temperature of ground beef.	2.40 ± 1.32	4.00 ± 1.11[d]	2.00 ± 1.44	3.87 ± 1.14[d]	2.20 ± 1.39	3.93 ± 1.12[d]
I determine the doneness of ground beef by its color[c]	2.20 ± 1.42	2.77 ± 1.14[d]	2.00 ± 1.05	2.40 ± 1.38	2.10 ± 1.24	2.58 ± 1.27[d]
I use a thermometer to ensure my refrigerator is set to the recommended temperature.	3.03 ± 1.40	4.23 ± 1.19[d]	2.27 ± 1.60	4.10 ± 1.35[d]	2.65 ± 1.54	4.17 ± 1.26[d]
I make sure my children wash their hands before meals.	4.63 ± 0.76	4.87 ± 0.43[d]	4.77 ± 0.50	4.97 ± 0.18	4.70 ± 0.65	4.92 ± 0.33[d]

[a]The statements for attitude, perceived behavior control, subjective norm, and behavior change intention were measure on a 5-point Likert scale: 1, strongly disagree; 5, strongly agree.

[b]The statements for self-reported safe food practice behavior were measured on a 5-point Likert scale: 1, never; 5, always.

[c]The statements "I cut meat and fruit on the same cutting board after rinsing with water."; and "I determine the doneness of ground meat by its color," were measured on a 5-point Likert scale: 1, always; 5, never.

[d]The mean scores between the pre- and post-survey for the construct's statement were significantly different, $P \leq 0.05$.
